# Liquid–liquid phase separation in tumor biology

**DOI:** 10.1038/s41392-022-01076-x

**Published:** 2022-07-08

**Authors:** Xuhui Tong, Rong Tang, Jin Xu, Wei Wang, Yingjun Zhao, Xianjun Yu, Si Shi

**Affiliations:** 1 Department of Pancreatic Surgery, Fudan University Shanghai Cancer Center, Shanghai, China; 2 Department of Oncology, Shanghai Medical College, Fudan University, Shanghai, China; 3 Shanghai Pancreatic Cancer Institute, Shanghai, China; 4 Pancreatic Cancer Institute, Fudan University, Shanghai, China; 5 Institutes of Biomedical Sciences, Shanghai Medical College, Fudan University, Shanghai, 200032 China

**Keywords:** Cancer genetics, Epigenomics

## Abstract

Liquid–liquid phase separation (LLPS) is a novel principle for explaining the precise spatial and temporal regulation in living cells. LLPS compartmentalizes proteins and nucleic acids into micron-scale, liquid-like, membraneless bodies with specific functions, which were recently termed biomolecular condensates. Biomolecular condensates are executors underlying the intracellular spatiotemporal coordination of various biological activities, including chromatin organization, genomic stability, DNA damage response and repair, transcription, and signal transduction. Dysregulation of these cellular processes is a key event in the initiation and/or evolution of cancer, and emerging evidence has linked the formation and regulation of LLPS to malignant transformations in tumor biology. In this review, we comprehensively summarize the detailed mechanisms of biomolecular condensate formation and biophysical function and review the recent major advances toward elucidating the multiple mechanisms involved in cancer cell pathology driven by aberrant LLPS. In addition, we discuss the therapeutic perspectives of LLPS in cancer research and the most recently developed drug candidates targeting LLPS modulation that can be used to combat tumorigenesis.

## Introduction

In the densely packed cellular space, the coordination of complex biochemical reactions in a spatial and temporal manner is important for performing biological function.^1^ Disruption of the precise spatiotemporal regulation can result in dysregulation of diverse cellular processes, including transcription,^2^ genomic integrity,^3^ chromatin organization,^4^ RNA processing^5^ and intracellular signaling.^6,7^ These processes are conceptual mechanisms underlying the complex hallmarks of cancer, including the loss of control over cell growth and proliferation, resistance to cell death, and metabolic reprogramming.^8^ Therefore, it is necessary to maintain normal spatiotemporal control in cells.

A possible cellular strategy for coordinating these reactions involves the formation of different compartments, where the density of the reaction components at a specified intracellular location can be adjusted.^9^ In fact, enzymatic reaction components are usually packaged in distinct subcellular compartments.^9^ For instance, classical organelles with a defined stoichiometry, such as the nucleus, Golgi apparatus, mitochondria and others, are lipid bilayer membrane-bounded compartments that make internal compounds inaccessible to almost all biomolecules and extracompartmental properties.

Notably, eukaryotic cells also achieve subcellular compartmentalization by forming a variety of nonstoichiometric biomolecular condensates without membranous structures,^10^ including promyelocytic leukemia (PML) protein bodies, nucleoli, paraspeckles, and Cajal bodies in the nucleus and stress granules (SGs), signaling puncta, and processing bodies (P bodies) found in the cytoplasm.^11–13^ Biomolecular condensates are composed of weak, multivalent interactions between macromolecules^9^ (for example, proteins and nucleic acids). In addition to the abovementioned punctate membraneless bodies, other subcellular structures also share similar physical properties, including heterochromatin^14^ and membrane receptor clusters at the cell membrane.^15^ The majority of these condensates exchange subunits rapidly with their surroundings within seconds or minutes.^16–18^

Increasing lines of evidence suggest that biomolecular condensates are reversibly and dynamically assembled via liquid–liquid phase separation (LLPS).^11^ LLPS is a physiological process that spontaneously drives the separation of a homogeneous solution of constituents into two or more coexisting phases: a dilute phase and a dense phase.^19^ Additionally, many studies have demonstrated that biomolecular condensates can be transformed into materials in different states, such as viscous liquids, gels, and even solid aggregates.^20–22^ The material features of biomolecular condensates are pivotal to distinct functions; for example, biocondensates form biochemical reaction centers, signaling hubs and the supporting architecture. Although cells have developed a variety of mechanisms to guarantee well-controlled LLPS, aberrant forms of phase separation are causatively related to many of the dysregulated cellular processes in cancer.^20^ In Table 1, we summarize the names, genes, localizations, structures and functions of 25 molecules related to the oncogenic mechanisms related to LLPS. For instance, the LLPS of NUP98-HOXA9 contributes to the formation of a broad super-enhancer (SE)-like binding pattern that potentiates the transcriptional activation of leukemogenic genes.^23^ Furthermore, it has been estimated that most cell signaling proteins, and even a large number of cancer-related proteins, have long intrinsically disordered regions (IDRs), which play critical roles in promoting LLPS.^24^

**Table 1:** Tumor-associated protein molecules that have been discovered to undergo LLPS

Protein	Organelle/Biomolecular condensates	Localization	Function	Role of phase separation in tumor
53BP1	Nuclear body	Nucleus	1. Respond to DNA damage.^126^ 2. Assists the tumor suppressor effect of p53.^131^	Hyper-accumulation of 53BP1 on chromatin and enhanced LLPS compromise cell survival in cancer cells.^199^
BRD4	Enhanceosome; nuclear body; nuclear bodies that occur at super enhancers in mESCs	Nucleus^114^	1. Epigenetic and transcriptional regulation.^310,311^ 2. Respond to DNA damage.^312^	The involvement of BRD4 in SEs plays a role in oncogene transcriptional addiction and cancer cell survival.^312^
CBX2	PcG protein complex; euchromatin; heterochromatin; PcG chromatin condensates^313^	Nucleus	1. Chromatin remodeling and modification of histones.^314^ 2. Promote the proliferation, invasion and migration of cancer cells.^315^	PcG protein complex can compact chromatin,^314^ and CBX2 is particularly upregulated in various cancers.^316^
DAXX	Nuclear body; nuclear protein granule; SPOP/DAXX body	Nucleus and cytoplasm	1. Transcriptional regulation. 2. DNA repair, and respond in viral infection. 3. Impact apoptosis and cell signaling.	SPOP/DAXX bodies formed via LLPS is important in inducing cancer cell apoptosis.^97,128^
SPOP	Nuclear body; nuclear protein granule; SPOP/DAXX body	Nucleus and cytoplasm	1. An E3 ubiquitin ligase substrate binding subunit of the proteasome complex that has both oncogenic and tumor-suppressive function in human cancers. 2. SPOP is frequently mutated in different cancers.^214,225,226,317^	Cancer-associated mutations of SPOP disrupt LLPS, and result in reduced protein ubiquitination.^215^
EWS	Nuclear protein granule^318^	Nucleus, cytoplasm and plasma membrane	1. Histone modification. 2. DNA methylation.^319^ 3. Fuse with transcription factor ELI.^320^	EWS-FLI1 specifically recruit BAF complex to activate target genes via binding to tumor-specific enhancers in Ewing sarcoma.^163^
HNRNPA1, HNRPA1	Cytoplasmic stress granule (SG)	Nucleus and cytoplasm	1. Forming SGs.^104^ 2. RNA splicing.^321^	SGs participate in apoptosis, immune modulation, and signaling pathways.^322^ SG assembly is aberrantly elevated in human cancers.^323^
HP1a	Heterochromatin^116^	Nucleus	1. Promote the formation of heterochromatin and plays a role in gene silencing. 2. Positively regulate gene transcription.^324–326^	Normal packaging and organization of heterochromatin is often compromised in cancer.^327^
MED1	Enhanceosome; nuclear body; nuclear bodies that occur at super enhancers in mESCs	Nucleus	1. A coactivator that involved in cancer-related transcriptional regulation and dysregulation.^328–330^ 2. Regulate autophagy.^331^	1. MED1 overexpression is associated with drug resisitance.^332^ 2. SEs mediate transcriptional addiction.^333^
NONO	Paraspeckle	Nucleus	1. It is required for mRNA splicing, DNA unwinding, transcriptional regulation, nuclear retention of defective RNA and DNA repair.^334^ 2. Induce cellular senescence. 3. Regulator of RNA:DNA hybrid related telomere instability.^335–337^	Paraspeckles influence the tumor stability to develop drugs resistance.^338^
SFPQ	Paraspeckle	Nucleus and cytoplasm	1. Regulate transcriptional activity, mRNA processing and splicing. 2. Regulator of RNA:DNA hybrid related telomere instability. 3. High SFPQ expression level in liver cancer is associated with cisplatin resistance.^337,339–341^ 4. DNA repair.	Paraspeckles influence the tumor stability to develop drugs resistance.^338^
NPM1	Nucleolus; granular component^342^	Nucleus	1. Cooperate with MYC to induce transcription of target proteins, thereby regulating the proliferation of normal cells and cancer cells. 2. It is mutated in acute myeloid leukemia. 3. NPM1 silencing cells play a role in migration and invasion ability.^343–347^	Larger nucleolar size and number are hallmarks of various cancers.^348^ Deregulation of nucleolar functions is correlated with tumorigenesis.^349^
NUP98	Nuclear pore central transport channel; selective hydrogel-like meshwork formed by FG-nucleoporins in nuclear pore central channel	Nucleus	1. Chromosomal translocations, changes in protein expression levels, and single point mutations. 2. Fuse with oncoproteins.^350,351^	NUP98-HOXA9 formed via LLPS induces leukemic transformation.^23^
PML	PML body	Nucleus and cytoplasm	1. Mediator of multiple apoptotic pathways. 2. A tumor suppressor. 3. Transcriptional regulation. 4. Regulate growth and invasion of cancer differentially.^352–354^	Disruption of PML bodies drives initiation of acute promyelocytic leukemia.^355,356^
YTHDF1, DF1	P-body; cytoplasmic stress granule;^357^ neuronal ribonucleoprotein granule	Cytoplasm	1. Higher expression in tumors than normal tissue in human cancers. 2. Regulate immune response and antigen processing and presentation.^358^ 3. mRNA binding, processing and degradation.^359^ 4. Regulate tumorigenicity and stem cell-like activity in cancer cells via Wnt/β-catenin pathway.^360^ 5. Overexpression of YTHDF1 promotes breast cancer progression.^361^	1. P-body-based regulation of mRNA metabolism plays an important role in cancer development and progression.^258^ 2. SG assembly is upregulated in cancer.^323^
YTHDF2, DF2	P-body; cytoplasmic stress granule; neuronal ribonucleoprotein granule	Nucleus and cytoplasm	1. mRNA binding, processing, stability and degradation. 2. YTHDF2 SUMOylation is important in post-transcriptional gene expression regulation and cancer progression.^362–365^	1. P-body-based regulation of mRNA metabolism plays an important role in cancer development and progression.^258^ 2. SG assembly is upregulated in cancer.^323^
YTHDF3, DF3	P-body; cytoplasmic stress granule; neuronal ribonucleoprotein granule	Cytoplasm	1. A key player in YAP signaling. 2. Translational regulation. 3. Higher expression in tumors than normal tissue in human cancers. 4. Overexpression of YTHDF1 and YTHDF3 promotes breast cancer progression.^359,361,366,367^	1. P-body-based regulation of mRNA metabolism plays an important role in cancer development and progression.^258^ 2. SG assembly is upregulated in cancer.^323^
TAF15	Nuclear protein granule	Nucleus and cytoplasm	1. mRNA binding, stabilization and regulation. 2. Stress response and DNA repair.^56^ 3. Translational control. 4. mRNA and protein levels of TAF15 are upregulated in liposarcoma.^368–370^ 5. TAF15 overexpression is associated with poor prognosis in patients NSCLC patients.^371^	Aberrant gene transcription through loci-specific phase separation, which contribute to oncogenic transformation ability in relevant cancers.^372^
P-TEFb	7SK snRNP complex, super elongation complex (SEC)	Nucleus	1. Elongation control of cellular transcription.^373^	SEC is a target for the mixed lineage leukemia (MLL) protein to activate the MLL target genes expression and promote leukemogenesis.^374^
OCT-4	MED1 droplets at SEs	Nucleus and cytoplasm	1. Cancer stem cells maintain expression of Oct4.^375^	SEs mediate transcriptional addiction in diverse cancers.^333^
YAP	YAP-TEAD complex/ YAP-TAZ-TEAD complex	Nucleus and cytoplasm	1. Transcriptional coactivator. 2. Effector of the Hippo signaling cascade. 3. Induce cancer stem cell attributes, proliferation, chemoresistance, and metastasis.^366,376^	YAP-TEAD complex is not only hyperactivated, but also confers a strong oncogenic activity in tumor tissues.^377,378^
TAZ	YAP-TAZ-TEAD complex	Nucleus, cytoplasm and plasma membrane	1. Transcriptional coactivator. 2. Effector of the Hippo signaling cascade. 3. Induce cancer stem cell attributes, proliferation, chemoresistance, and metastasis.^366,376,379^	YAP-TAZ-TEAD complex is not only hyperactivated, but also confers a strong oncogenic activity in tumor tissues.^377,378^
DDX3	Cytoplasmic stress granule	Nucleus, cytoplasm and plasma membrane	1. ATP-dependent RNA helicase involved in DNA repair.^380^ 2. Transcriptional regulation. 3. Initiate and regulate translation.^381^ 4. Involved in stress and inflammatory responses.^382^	Cancer-associated mutations of DDX3X cause SG hyper-assembly and translation impairment.^383^
HSF1	HSF1 nuclear stress bodies (HSF1 foci)	Nucleus	1. Transcriptional regulator of chaperones. 2. Cancer cell invasion, proliferation, and metabolism.^384^	HSF1 foci are preferentially located in cancer cells of primary human tumors.^384^
ENL	Nuclear protein granule	Nucleus	1. Frequently fuse with the MLL protein and resultant fusion proteins function as oncogenic drivers in acute myeloid leukemia (AML) and acute lymphoid leukemia (ALL).^385^	ENL LLPS enhance recruitment of SEC and drive transcription.^386,387^

In this review, we describe recently obtained insights and findings on the formation, dynamics, regulation, and function of LLPS and highlight the various effects of biomolecular condensates on cell biology. Our review specifically focuses on the dysregulated LLPS that occurs in aberrant cellular processes associated with carcinogenesis, including chromatin organization, epigenetics, oncogenic transcription, aberrant signaling pathways, and telomere lengthening mediated by LLPS. Clinical evidence for the occurrence of abnormal LLPS processes in cancer patients has also been extensively collected, and this information provides a substantial basis for the importance of LLPS in tumor biology. For example, Meng et al. observed phase-separated droplets formed by Merlin (NF2) in dissected samples from vestibular schwannoma patients.^25^ In addition, emerging evidence suggests that biomolecular condensates affect the pharmacodynamic properties of antineoplastic medicines; therefore, regulating the LLPS process could be a potential strategy for novel cancer therapies. Therefore, we evaluate the great potential of effectively regulating LLPS in anticancer therapy and propose perspectives on condensates that might contribute to future investigations in oncology.

## Liquid–liquid phase separation

LLPS is a decent explanation for the formation of multiple membraneless structures in cells.^26^ The profound exploration of membraneless organelles began with the discovery of P granules in *Caenorhabditis elegans* in 2009. The P granules are liquid-like structures composed of proteins and RNAs, which can flow, fuse, deform and fission under shear force.^27^ Following the study of P granules, other intracellular substructures, such as the nucleoli, SGs, and paraspeckles, have also been revealed to be formed by LLPS and are enriched in RNA-binding proteins (RBPs) and RNAs.^28,29^

LLPS is thought to be triggered by weak, multivalent interactions between proteins and nucleic acids^13^ rather than covalent, high-affinity interactions. These weak interactions also play a pivotal role in concentrating components at discrete cellular sites,^30^ which is very important to ensure the accurate spatiotemporal regulation of normal cell biological activities. Below, we summarize the thermodynamic conditions necessary to trigger LLPS and various interactions between proteins and nucleic acids that promote LLPS.

### Thermodynamic conditions of LLPS

If we consider the cytoplasm or nucleoplasm as a macromolecular solution, the surface of macromolecules would exhibit weak, transient, nonspecific interactions with each other and with the solvent.^31^ These low-affinity interactions tend to dissolve the molecules and evolve the entire system into a well-mixed state (entropy).^31^ The solubility of macromolecules is dominated by the balance of weak interactions between macromolecules and macromolecules versus macromolecules and solvent.^32^ As the concentration of macromolecules in the solution is increased to the solubility limit—the threshold concentration—the interactions between the macromolecules will become stronger than the interactions between the macromolecules and the solvent, and as a result, this solution will gain propensity to LLPS.^33^ The threshold concentration depends on several biophysical parameters, such as the salt concentration, temperature, and other ions.^34^ Therefore, the concentration dependence suggests that the threshold concentrations are hallmarks of LLPS.^19^

### Multivalency driving LLPS

The macromolecules undergoing LLPS can be classified into scaffolds and clients.^35^ Scaffold molecules are characterized by multivalent proteins or nucleic acids that are indispensable for triggering LLPS.^36^ Higher valency enables the formation of larger oligomers or polymers at lower saturation.^37^ Multivalency can be achieved by proteins containing multiple folded domains, IDRs,^28^ and nucleic acid chains.^38^ The client proteins are molecules with low valency that are recruited by the scaffold and partition into condensate structures. Clients cannot themselves undergo LLPS, and their recruitment level is determined by the scaffold stoichiometry.^35^

Compared with the very-low-affinity interactions determining solubility, the multivalent interactions between macromolecules show higher affinity and high stereospecificity and enable assembly into large oligomers or polymers.^39^

#### Multiple folded domains that promote LLPS

**Fig. 1:**
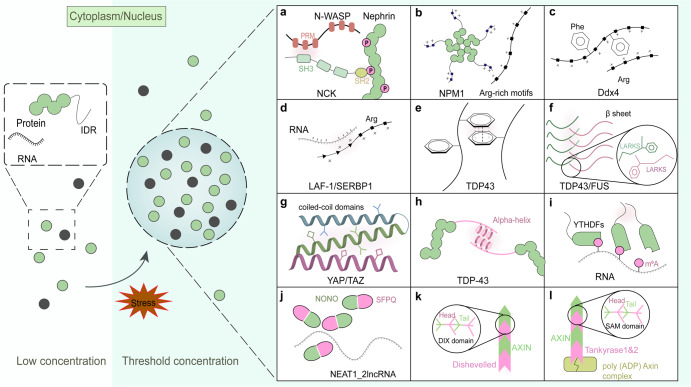
Summary of various types of interactions that promote the occurrence and maintenance of LLPS. **a** Nephrin contains three phosphotyrosines (pTyrs) motifs, which bind the SH2 domain of Nck, and Nck possesses three SH3 domains that can bind the ~six proline-rich motifs (PRMs) of N-WASP. **b** The unphosphorylated form of the Npm1 monomer oligomerizes into pentamers through its N-terminus (Npm-N) and binds to proteins with Arg-rich linear motifs. **c** In Ddx4, Phe and Arg motifs in the intrinsically disordered regions (IDRs) drive LLPS. **d** In LAF-1 and SERBP1 proteins, the positively charged Arg/Gly-rich (RGG/RG) domain binds to negatively charged RNA and effectively promote LLPS. **e** In TDP43, the pi-pi interactions in IDRs facilitates LLPS. **f** Low-complexity amyloid-like reversible kinked segments (LARKS) in TDP43, and FUS proteins would give rise to phase separation via mediating reversible amyloid-like interactions. **g** Hydrophobic interactions existing in coiled-coil domains are essential for phase separation. **h** Local α-helix in the C-terminus of the TDP-43 protein enables TDP-43 self-connections and facilitates LLPS. **i** LLPS of YTHDF1 can be enhanced by the mRNAs with multiple m6A residues. **j** NEAT1_2 lncRNA subdomains selectively bind NONO/SFPQ proteins and dynamically oligomerize. **k** In the Wnt signaling cascade, Disheveled contacts the DIX domain of the AXIN protein in a head-to-tail manner and transduce Wnt signals into the nucleus. **l** SAM domains of tankyrase protein form dynamic puncta via head-to-tail polymerization with AXIN, promoting Wnt signaling

LLPS in living cells was initially investigated based on the multivalent interactions among Nephrin, Nck and neural Wiskott-Aldrich Syndrome protein (N-WASP). The phosphotyrosines (pTyrs) in Nephrin can each bind the SH2 domain of Nck, and Nck possesses three SH3 domains that can bind the ~six proline-rich motifs (PRMs) of N-WASP (as shown in Fig. 1a).^37^ When the adhesion receptor Nephrin is attached to the lipid bilayer, the three-component system (Nephrin/Nck/N-WASP) can also undergo LLPS and form liquid-like clusters on the membrane surface.^40^ Similarly, the signaling proteins governing the organization of actin in T cells can also assemble into membrane puncta in response to T-cell receptor (TCR) activation.^15^

Another example is Nucleophosmin (Npm1), a highly abundant protein that contributes to the maintenance of genome stability and regulation of the p53 tumor suppressor pathway and has been implicated in mediating the LLPS of nucleolar granule components.^41–43^ The Npm1 monomer encodes only a 2-valent interaction partner, which is insufficient for triggering phase separation. However, the unphosphorylated form of the Npm1 monomer oligomerizes into pentamers through its N-terminus (Npm-N) and binds to proteins with Arg-rich linear motifs. The oligomerizing process effectively increases the valency to 10, which is sufficiently high to mediate phase separation (Fig. 1b).^44,45^ This excellent example proves the importance of multivalency between interacting motifs for controlling LLPS. Similarly, the speckled POZ protein (SPOP), a tumor suppressor, is a cullin-3-RING ubiquitin ligase (CRL3) substrate adapter that can self-assemble into higher-order polymerized forms and localize to nuclear speckles.^46^

#### IDRs promote LLPS

The IDRs in proteins are another method for gaining multivalency and driving LLPS. IDRs lack a stable tertiary structure, which allows access to a wider conformational space and enables the formation of three-dimensional networks of protein molecules.^47^ These regions have low amino acid sequence complexity and comprise only a limited set of amino acid types, such as Gly, Ser, and Gln, and aromatic residues, including Phe and Tyr.^9,48,49^ Recent evidence suggests that the aromatic residues in IDRs are particularly important for the promoting effect. In Ddx4, the cation-pi interactions between Phe and Arg motifs have been proven to be significant for driving LLPS (Fig. 1c).^50^ Analogously, the Tyr residues in other RBPs, such as fused in sarcoma (FUS),^51^ hnRNPA1^52^ and BuGZ,^53^ can also promote the process of phase separation in vitro and/or in cells. The mutation of Tyr residues in BuGZ^53^ and FUS^54^ significantly blocks the formation of phase-separated liquid droplets.

**Fig. 2:**
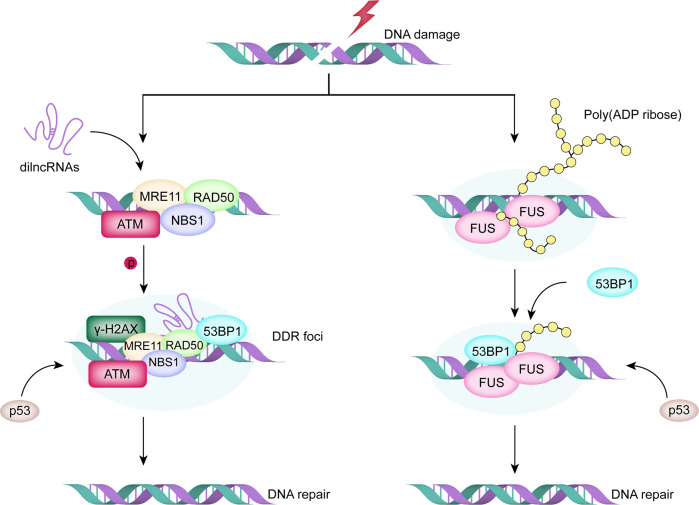
LLPS supports various DNA damage response (DDR) mediator to form DNA repair foci in different pathways. (Left) MRE11–RAD50–NBS1 complexes bind to the exposed DNA damage sites to initiate the pathway. Subsequently, the damage-induced lncRNAs (dilncRNAs) and P53-binding protein 1 (53BP1) are recruited to the DDR site to promote the formation of DNA damage repair foci via LLPS. (Right) Nucleation of PAR with FUS and 53BP1 at DDR sites forms liquid-like compartments and facilitates subsequent signaling and repair

Additionally, the electrostatic interactions (salt bridges) between opposing charge residues contribute to the promotion of LLPS. In the Caenorhabditis elegans protein LAF-1, the positively charged Arg/Gly-rich (RGG/RG) domain binds to negatively charged RNA and effectively promotes the formation of P granules (Fig. 1d).^55^ The same interaction pattern can also be observed in nucleation events at DNA damage response (DDR) sites, where negatively charged poly(ADP-ribose) (PAR) can rapidly recruit positively charged proteins containing IDRs and cause liquid demixing upon DNA damage (Fig. 2).^56^ Moreover, IDRs can drive LLPS through other weak interactions, such as pi-pi interactions mediated by aromatic residues (Fig. 1e),^57^ reversible amyloid-like interactions occurred in TDP43, and FUS proteins (Fig. 1f),^58,59^ hydrophobic interactions triggered by coiled-coil domains (Fig. 1g),^60–62^ along with dipolar interactions (data was not shown).^63^

In addition, the short stretches of amino acids in IDRs can form localized structures and promote self-interaction. For instance, the IDRs located in the C-terminus of the TDP-43 protein contain a structural domain that forms a local α-helix, which enables TDP-43 self-connections and facilitates LLPS (Fig. 1h). Mutations affecting the structure or intermolecular contacts in the TDP-43 alpha-helix and/or nearby regions can disrupt multivalent interactions and result in altered phase separation.^64^

#### RNAs modulate phase behavior

As mentioned above, RNAs are found in membraneless condensates^19,20,65^ and have been shown to promote LLPS.^65^ RNAs can not only drive LLPS via electrostatic interactions, but repetitive intermolecular base pairing can also achieve multivalency and thus drive the formation of clusters in vitro and in vivo.^65^ For example, RNAs added to SERPINE1 mRNA-binding protein 1 (SERBP1) medium effectively induce LLPS by interacting with the RG/RGG-rich domains of SERBP1 (Fig. 1d). Furthermore, fluorescence signals are more rapidly recovered in the presence of RNA, which suggests that RNA at a certain concentration (0.05 mg/ml) (measurement of the RNA sequence 5’-GCGCGGG-3’) makes SERBP1 droplets more dynamic and fluid.^66^ However, the experiments conducted by Burke and Janke suggest that FUS monomers can interact with RNA to initiate the construction of fibrillar FUS condensates, but a higher quantity of RNA dissolves FUS condensates.^51^

N6-methyladenosine (m^6^A) is the most frequent nucleotide modification of mRNA^67,68^ and is necessary for various cellular and physiological processes. The expression level of the correlated proteins is frequently increased in a variety of human cancers.^69,70^ In diverse RNP granules, including SGs, keratin granules, and P-bodies, the m^6^A-binding protein YTHDFs in the cytoplasm undergo spontaneous LLPS in vitro and in extracted cells, and the process can be clearly enhanced by the addition of mRNAs containing multiple m^6^A residues (Fig. 1i).^68^ Polymethylated mRNAs may function as scaffolds for proteins with multivalency in combination with YTHDF proteins through their IDRs, resulting in LLPS.^68^

In addition, a great number of long noncoding RNAs (lncRNAs) are localized on chromatin and often form an RNA cloud in a particular nuclear area to regulate gene expression. ncRNAs are major components of several membraneless structures, such as the nucleolus and paraspeckles,^54,71^ the formation of which is frequently dysregulated in cancers. Numerous findings have recently demonstrated that NEAT1_2 lncRNA subdomains can selectively attach to NONO/SFPQ proteins, and these proteins can dynamically oligomerize and recruit additional proteins through LLPS to facilitate the assembly of paraspeckles (Fig. 1j).^72^

#### New mechanism to multivalency—head-to-tail polymerization

In contrast to IDRs, there remain stably structured domains in proteins that can attain multivalency by improving local condensation.^73^ The DIX and SAM domains are two distinct domains capable of spontaneously assembling dynamic head-to-tail polymers, condense into filaments and are then crosslinked to form three-dimensional condensates.^74,75^ The DIX domain was discovered in the Wnt signaling cascade, Disheveled,^76^ which can contact the DIX domain of the AXIN protein in a head-to-tail manner and transduce Wnt signals into the nucleus (Fig. 1k).^77^ Analogously, the SAM domains of tankyrase protein can also form dynamic puncta via head-to-tail polymerization, promoting Wnt signaling by binding to and ribosylating the poly (ADP) AXIN destruction complex (Fig. 1l).^78^ The difference between the DIX and SAM domain interactions is that the affinities of SAM domains are higher than those of DIX domain-mediated interactions.^74^

### Physiological regulation of LLPS

Any factor that influences the properties of the condensate component affects the condensate structure, dissolution, viscoelasticity, and other physicochemical features that influence their functions,^12,38^ such as protein concentration, posttranslational modifications (PTMs) or environmental elements, providing a vigorous mechanism for the regulation of cells.^79^

#### Biophysical parameters

The concentrations of biomolecular components are essential factors in condensate formation and dissolution. Expanding the volume of nucleoli by placing them into a hypotonic solution can cause reversible solubilization of PML bodies and nucleoli as the matrix is diluted.^80^ A variety of pathways influence the concentration, such as influencing biosynthesis, degradation, transportation, and localization.^38^

Because of the importance of thermodynamics, for some of the molecules that reach their dissolution limit, even a slight disturbance to physical parameters, such as a concentration or temperature chance, can induce rapid phase changes. For instance, a change of 1 °C may lead to BUgZ, DDX4, or FUS droplets condensing or dissolving.^9^ The prion-like domains of these proteins can sense pressure regulated by the environment, which in turn influences the solubility and phase behavior of the protein.^81^ For example, TDP43 and FUS cluster to SGs under physiological stress conditions such as heat and oxidative stress.^82,83^

Other biophysical elements, such as the salt concentration in the milieu^66^ and the addition of PEG3000 and glycerol, can also effectively regulate LLPS.^66^

#### Posttranslational modifications

Emerging evidence suggests that PTMs, which include phosphorylation, acetylation, arginine (Arg)-methylation, and SUMOylation, play pivotal roles in regulating phase separation.^84,85^ The downstream reactions of cells to various stimuli are often influenced by PTM-triggered signaling.^86^ PTMs induce a wide range of effects on the structural properties of intrinsically disordered proteins and potentially drive complete state changes among different states, such as intrinsically disordered states, folded states, dispersed monomeric, and phase-separated states.^87^ In LLPS, PTMs can alter the physicochemical characteristics of the regulated amino acids in scaffold proteins, such as by changing their valency, electric charge, or volume.^35^ PTMs can also influence the interactive conditions to affect phase separation, such as by directly diminishing or enhancing the multivalent interactions between macromolecules, recruiting certain macromolecules into the condensate or excluding macromolecules from the condensate.^84^ For instance, the Arg residues of RGG/RG motifs in FUS are largely modified by the deposition of asymmetric dimethyl groups by protein arginine methyltransferase (PRMT) 1 or 8,^88–90^ which in turn decreases the LLPS rate of FUS and enhances condensate dynamics.^91^ An overabundance of Arg methylation mediated by PRMT1 increases the rate of cytosolic FUS and SG partitioning in response to oxidative stress.^82^ Additionally, Arg methylation reduces hnRNPA2 phase separation and destabilizes the Ddx4 droplets by diminishing Arg-aromatic(pi) interactions.^50,92^

In addition, another PTM called SUMOylation involves the covalent attachment of small ubiquitin-like modifiers (SUMOs) that modulate cellular processes in the nucleus.^93^ Lys residues in disordered regions have been found to be the preferred target of SUMOylation, different from other common Lys PTMs.^94^ SUMOylation in PMLs contributes significantly to the formation of the PML nuclear body, whereas de-SUMOylation can lead to a constituent protein being released and nuclear bodies being separated during mitosis.^50,95^ In addition, death domain-associated protein (DAXX) possesses highly conserved SUMO-interacting motifs,^96^ which are needed for DAXX linkage with SUMOylated PML oncogenic domains, and its expression is upregulated in multiple cancers.^97^ DAXX can bind to SUMOylated SMAD4 and suppress SMAD4-mediated transcription.^27^ SMAD4 is activated downstream of the cellular effects of TGF-β, which induces apoptosis and prevents proliferation, and the loss of SMAD4 expression potentiates tumorigenesis,^93,98^ which suggests the potential significance of DAXX in carcinogenesis. In addition, SUMOylation of SERBP1 is thought to be a trigger for glioblastoma multiform progression because aberrant SUMOylation pathways may result in cancer progression.^99^

Furthermore, it has been demonstrated that in response to DNA damage, the N-terminus of FUS is phosphorylated by DNA-dependent protein kinase (DNA-PK), and this phosphorylation leads to FUS translocation from the nucleus to the cytoplasm. The translocation is mediated by the phosphorylation of serine or threonine residues on the N-terminus of FUS by DNA-PK, but the exact mechanism remains uncertain.^100^ Additionally, Ding et al. revealed that the phosphorylation of Ser61 occurs specifically at the Ser61 site of FUS, which can effectively disrupt the intra- and intermolecular interactions that maintain pathological aggregation in cells.^101^ Therefore, we believe that diverse PTMs can be an effective approach for regulating the process of phase separation and possibly affect oncogenic processes.

#### ATP and phase separation

The role of ATP in LLPS has thus far been subject to considerable uncertainty. First, ATP prevents RNA/proteosome assembly while maintaining protein solubility.^102,103^ A high concentration of ATP inhibits the tendency of IDRs in granular components to assemble into stable amyloid fibrils.^104,105^ Brangwynne et al. also found that the liquid-like properties in the spherical state of the nucleolus depend on ATP, which suggests that the nonspherical nucleolar profile may indicate changes in metabolism.^106^ However, a range of evidence confirms that ATP promotes the formation of nuclear condensates.^107^ Therefore, the exact role played by ATP in LLPS remains difficult to discern, but we look forward to more ideas from future researchers.

#### Chaperones and phase separation

Molecular chaperones are essential components of the quality control system in cells to sustain protein homeostasis (proteostasis) and thus avoid aberrant folding and aggregation.^108^ A wide spectrum of molecular chaperones has been proven to undergo LLPS, including heat shock protein 27 (Hsp27), class I and II Hsp40, Hsp70, and Hsp90, and most of chaperones have been found to be incorporated into SGs.^109,110^ Liu et al. demonstrated that small Hsp27 prevents LLPS of FUS protein by interfering with intra- and intermolecular transient interactions of the low-complexity domain in FUS. However, the phosphorylation of Hsp27 induced by cellular stress conditions, including sodium arsenite, heat shock, or oxidative stress, reduces its inhibitory function.^110^ Analogously, in neuron terminals, transportin also functions as a chaperone of FUS proteins, inhibiting the phase separation process and SG partitioning.^111^

In patients with fibrolamellar carcinoma, loss of myristylation and gain of Hsp70 binding by oncogenic DnaJB1-PKA_cat_ are responsible for abolishing RIα LLPS. The DnaJB1-PKA_cat_ fusion oncogene has been detected in nearly all fibrolamellar carcinoma patients, and the inhibition of RIα LLPS results in disruption of cAMP compartmentation and deregulation of the cAMP/PKA signaling pathway, which consequently leads to tumorigenesis.^112^ Therefore, the above-described example illustrates the importance of normal phase separation to the organism and the pathogenic potentiality of molecular chaperones in the regulation of LLPS.

### Functions of biomolecular condensates

LLPS is involved in multiple biological processes in cells, such as chromatin architecture, DNA damage repair, transcriptional regulation, intracellular signaling, and protein degradation.^113^ These activities take place throughout all types of cells, and abnormalities in these processes are central events in the study of tumor biology. Therefore, we reasoned that LLPS might contribute to tumor pathogenesis and tumor progression.

#### LLPS in chromatin organization

LLPS of chromatin-associated factors can promote the organization of the chromatin structure to regulate transcription.^14,114,115^ Gibson et al. found that reconstituted chromatin undergoes LLPS both in vitro and in cells.^4^ Recent studies suggest that heterochromatin protein 1 (HP1) can form liquid-like droplets via phase separation.^14,116^ Heterochromatin is a tightly packaged chromatin structure that is an important component of eukaryotic genome sequences and is pivotal for the normal organization of chromosomes, genome integrity, DNA replication,^117^ transposon silencing and gene expression.^118^ A prominent characteristic of heterochromatin is trimethylation of Lys 9 on histone H2 (H3K9me2) and H3K9me3.^119^ Some findings have revealed that LLPS specifically takes place at transcriptionally active regions of DNA with lower chromatin density. Shin et al. revealed that phase-separated liquid condensates are preferentially formed at chromatic regions with low density and mechanically push out nontargeted chromatin. Thus, distant targeted genomic loci can be mechanically pulled together and restructured through the fusion of droplets.^114^ The causative linkage between LLPS and chromatin compression has been demonstrated in an increasing number of studies.^120^ DNA demethylation in heterochromatin has been linked to chromosome translocations in different types of cancer, such as breast, lymphoid, and endothelial tract cancer.^121,122^ Additionally, reduced hypermethylation of heterochromatin has been highly correlated with human cancer progression and metastasis.^123,124^

#### LLPS in DNA damage repair

The DDR is responsible for safeguarding genomic integrity and stability, whereas defects in the DDR may result in oncogenic mutations.^125^ In response to DNA damage, LLPS supports the process of various DDR mediators forming repair compartments in two different ways (Fig. 2).^126^ Upon DNA damage, MRE11–RAD50–NBS1 complexes can recognize and bind to the exposed DNA damage sites and recruit damage-induced lncRNAs (dilncRNAs). dilncRNAs then attract the tumor suppressor P53-binding protein 1 (53BP1) to the DDR site and promote the formation of DNA damage repair foci via LLPS (Fig. 2 left).^127^ This method of repairing DNA double-strand breaks is very common in cancer cells and results in the avoidance of apoptosis.^128^

The earliest cellular response to DNA lesions in another pathway starts with the nucleation of PAR with multiple intrinsically disordered proteins, including FUS, at DDR sites.^127,129^ FUS is precisely directed to DNA damage sites by long and branched PAR chain formation.^130^ After the PAR signal terminates, 53BP1 gains access to the DNA damage sites, forms liquid-like compartments and facilitates subsequent signaling and repair (Fig. 2 right).^131^

#### LLPS in transcriptional regulation

As a critical step in gene expression, dysregulated gene transcription can initiate the uncontrolled proliferation of cancer cells.^132^ Interestingly, emerging evidence demonstrates that LLPS plays an important role in the progression of transcription and RNA processing.^133,134^ For example, the C-terminal domain (CTD) of the RPB1 subunit of human RNA polymerase (Pol) II is composed of a highly repetitive, unstructured protein domain of low complexity.^135^ The FUS, Ewing sarcoma (EWS), and TAF15 genes can form a liquid-like phase-separated state, directly bind the CTD of RNA Pol II and activate transcription.^51,136^ Furthermore, recent evidence demonstrates that transcription factors (TFs), RNA Pol II, chromatin regulators and various coactivators aggregate into condensates (called SEs)^137^ via phase separation at those highly transcribed genes.^138^

**Fig. 3:**
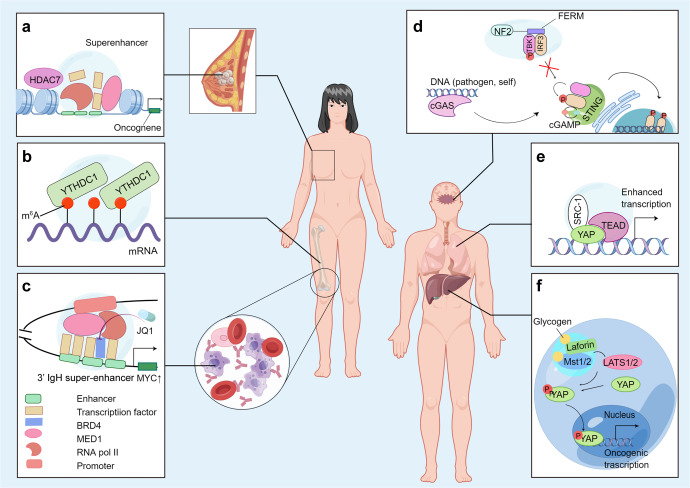
The roles of phase separation in various cancers. (By Figdraw.). **a** In stem-like breast cancer cell model, the histone deacetylase HDAC7 binds near the transcriptional start site and to SEs of various oncogenes. **b** YTHDC1 undergoes LLPS via binding with m^6^A-mRNA, and the number of resulting nuclear condensates (nYACs) is greatly increased in acute myeloid leukemia (AML) cells. **c** In multiple myeloma (MM) cells, the 3’ IgH super-enhancer (SE) inserts near the MYC locus, driving the upregulation of MYC expression. **d** Mutant FERM domain of NF2 form phase-separated condensates with IRF3 and abrogates the antitumor immunity initiated by STING. **e** In lung cancer, the liquid-like YAP/TEAD/SRC-1 compartments in nucleus can broadly upregulate YAP transcription. **f** Glycogen accumulation and phase separation lead to the formation of Laforin-Mst1/2 complex, thus activate oncogenic YAP signaling

In addition, strong evidence demonstrates that the transcriptional coactivator Yes-associated protein (YAP)^139^ and the transcriptional effector PDZ-binding motif (TAZ)^140^ compartmentalize the transcriptional cofactors and coactivators to facilitate the expression of target genes by LLPS.^62^ In lung cancer, the YAP-generated condensates can further form YAP/TEAD/SRC-1 compartments by interacting with SRC-1 and extensively improve YAP transcription (as shown in Fig. 3e).^141^ It is well known that YAP and TAZ are downstream effectors of the Hippo pathway—a tumor suppressor signaling pathway that is important for biological activities such as immune regulation, epithelial homeostasis, and tissue regeneration.^142^ Therefore, aberrant YAP/TAZ-mediated transcriptional condensates may contribute to cancer-related pathophysiology.

Furthermore, intracellular phase-separated liquid-like structures regulate the localization and processing of mRNA.^143,144^ For example, AKAP95, a nuclear protein that participates in RNA splicing, generates liquid-like phase-separated condensates in vitro and in cells.^145^ AKAP95 is frequently overexpressed in human breast cancer, and the liquid condensates possess the abilities to support tumorigenesis with proper liquidity and dynamicity,^145^ which indicates that modifying the properties of biomolecular condensates can potentially target LLPS and provide useful ideas for cancer therapy. The mechanisms of RNA distribution and processing in cells are important for subsequent protein localization and function, which implicates the multistep nature of gene expression regulation.^146^

#### LLPS and intracellular signaling

Various signaling transduction pathways have been implicated in the regulation of cell proliferation, differentiation, metabolism, angiogenesis, apoptosis and senescence.^147,148^ Emerging evidence suggests the importance of phase separation in orchestrating signaling pathways through the compartmentalization of significant factors.^149^ For example, when TCR phosphorylation is triggered, the downstream signaling proteins also form liquid-like clusters spontaneously via LLPS, which results in the promotion of signal outputs.^15^

**Fig. 4:**
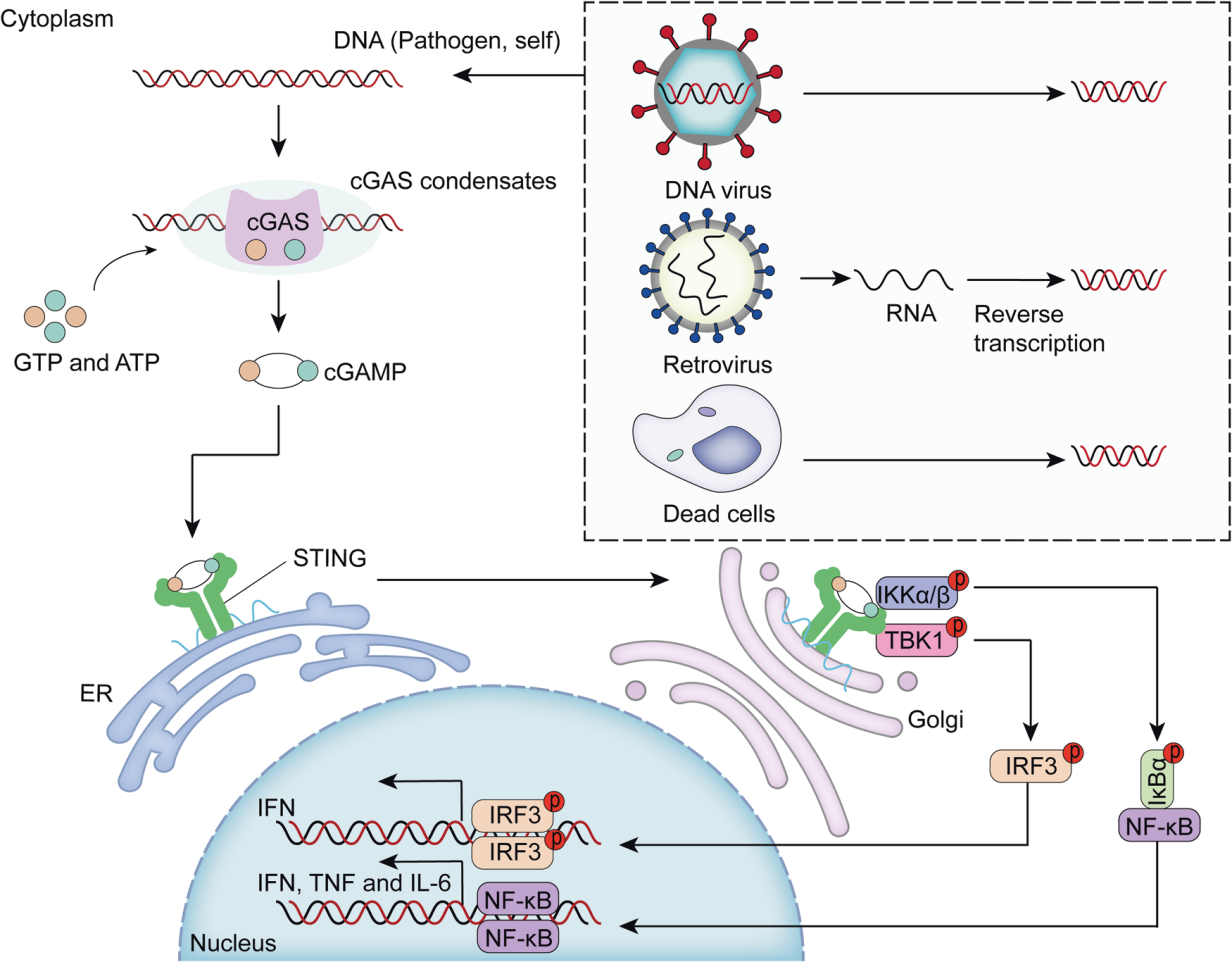
Phase separation of enzyme cyclic GMP-AMP synthase (cGAS) contributes to innate immunity. The cellular enzyme cGAS directly induces phase separation via binding with DNA and produces cGAMP. cGAMP interacts with STING and activates downstream innate immunity

Furthermore, the cellular enzyme cyclic GMP-AMP synthase (cGAS) functions as a direct DNA sensor and produces the secondary messenger cyclic GMP-AMP (cGAMP), which has an innate immune function (Fig. 4).^150^ Interactions with DNA induce phase separation of cGAS and promote the production of cGAMP, which interacts with the receptor stimulator of interferon genes (STING), activates downstream type I interferon and NF-κB signaling^151^ and facilitates innate immunity.^150^ Therefore, normal and properly regulated LLPS is essential for the human body to maintain normal immune signaling pathways, and if the LLPS process is aberrantly changed, it is likely to cause pathological consequences, such as cancer.

#### LLPS and autophagy

Phase separation is also involved in autophagy.^20^ Autophagosome formation involves the process of LLPS at specific sites, and LLPS can positively regulate autophagy activity through different mechanisms.^152^ Specifically, phase separation plays a role in modulating TORC1 activity.^153^ TORC1 is a Ser/Thr kinase complex that regulates multiple cellular processes and cellular metabolism in response to nutrient availability in the milieu.^154^ The inhibition of TORC1 signaling can induce autophagy, and dysregulated TORC1 and autophagy have been demonstrated to be correlated with tumorigenesis.^155^ Mechanistically, LLPS of yeast Pbp1 in combination with a cellular redox state can lead to TORC1 downregulation and consequently promote autophagy.^155^ In addition, LLPS plays a role in forming aminopeptidase Ape1 condensates, which are trafficked by double-membrane-bound Cvt vesicles and thereby enable selective engulfment.^156^

Furthermore, biomolecular condensates can control protein quality by not undergoing autophagic degradation.^157^ For example, stress-sensitive proteins in the nucleus misfold under pressure and then aggregate into the nucleolus after being in a nucleoplasmic dispersion state. After combining with nucleolar proteins through LLPS, these misfolded proteins are protected from irreversible aggregation,^158^ which facilitates refolding during recovery from stress.

In summary, LLPS is important to a variety of cellular processes, as explained herein. Therefore, we will discuss the possible oncogenic effects of various aberrant LLPS and the states of LLPS processes in tumor cells of different cancer patients.

## Aberrant LLPS in cancer

As described previously, biomolecular condensates are involved in the control and regulation of various cellular biological processes, and the constituent macromolecules of biomolecular condensates are affected by genetic abnormalities in various cancers, which suggests that condensates are significant for unraveling the carcinogenesis process and prompting new advancements in cancer therapy. Malignant cells acquire genomic mutations that influence various biological processes mediated by LLPS during tumorigenesis, including chromatin changes, transcription, DNA damage repair, and tumor suppression. These mutations may result in aberrant cellular activity, such as driving unlimited proliferation and replicative immortality, angiogenesis, cancer cell evasion from growth suppressors, resistance to death, invasion and metastasis.^3,132^ Furthermore, recent studies have demonstrated that aberrant LLPS can influence epigenetic regulation, which is also associated with the onset and progression of cancers. Therefore, we suspect that LLPS may provide a useful framework for understanding the emergence and progression of diverse cancers and for ultimately finding appropriate treatments.

### The deregulation of LLPS in chromatin organization contributes to tumorigenesis

A high frequency of mutations in genes encoding the elements modulating chromatin architectures has been found in numerous cancers.^159–161^ Genetic alterations of the components of phase-separated droplets regulating chromatin organization have also been found in recent studies.^162^ For instance, the chromatin remodeling complex BRG1/BRM-associated factor (BAF) is recruited by the EWS-FLI1 chimeric protein to activate oncogenic gene expression in Ewing sarcoma.^163^ The BAF complex mediates chromatin remodeling in an ATP-dependent manner, and cells with mutated BAF subunits show damaged chromatin structures and are unable to express multiple genes.^164^ The IDRs in EWSR1 effectively mediate strong interactions with FLI1 and form liquid-like compartments, which is significant for the tumorigenicity of Ewing sarcoma,^163,165^ and the genes encoding BAF complex subunits are also frequently mutated in different cancers.^166^ To develop therapeutic agents for Ewing sarcoma, Martin et al. extensively summarized various agents and factors affecting EWS-FLI1 activity, such as hypoxia, miRNAs, and antibodies against the IGF-1/IGF-1R pathway.^165^

### SEs drive oncogenic transcription

**Fig. 5:**
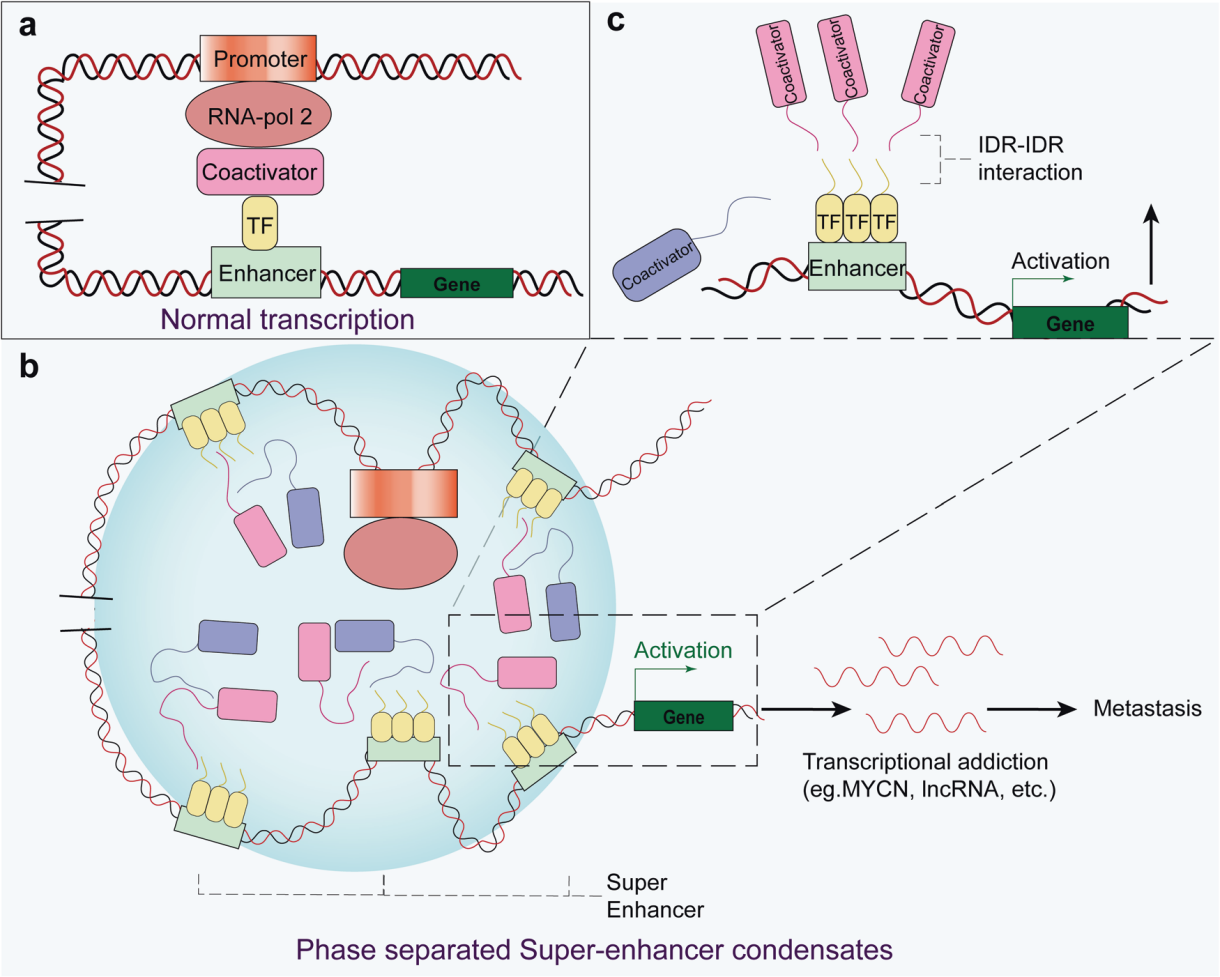
Components and processes in normal transcription and SE-mediated transcriptional addiction of genes in cancer. **a** The normal transcription of a gene involves proper interactions among the promoter, RNA pol 2, coactivators, TFs and enhancers. **b** The SE condensates form a liquid-like complex via LLPS and may result in the transcriptional addiction of certain genes. **c** Detailed illustration of the IDR-IDR interaction that promotes SE formation

Abnormal gene transcription driven by mutations in the genetic and epigenetic landscape contributes to the initiation of uncontrollable growth and proliferation of cancer cells.^132^ Different from normal transcriptional pathway (Fig. 5a), the activation of prominent oncogenes and other genes related to tumor pathogenesis is possibly induced through SEs, which comprise several hundred clusters of enhancer elements, TFs and enhancer-related modifications that regulate gene transcription important to different cell types (Fig. 5b, c).^137,167^ These genes are particularly sensitive to the disruption of oncogenic signaling pathways.^168^ Emerging evidence suggests that TFs containing IDRs, transcription coactivators and RNA pol II form phase-separated condensates at SEs^169^ and that the protein and nucleic acid components of SEs are subject to phosphorylation; SEs bind proteins based on their phosphorylation status.^137^

Researchers have discovered that Epstein–Barr virus (EBV) nuclear antigen 2 (EBNA2) and its coactivator EBNALP undergo LLPS mediated by their IDRs at active SEs by interacting with numerous TFs, which promotes their own transcription.^170^ EBV is one of the most significant human tumor viruses and is correlated with various cancers, including nasopharyngeal carcinoma, gastric cancers, and Hodgkin lymphoma.^171–175^ Malignant cells may acquire SEs via diverse mechanisms, including genomic rearrangements, focal amplifications of SEs commonly associated with other genes, and highly asymmetric loading of oncogenic TFs.^176–178^ SE acquisition through these mechanisms is related to a large number of oncogenes that are being currently studied.^176,179,180^ Notably, the protein products of these genes are important for controlling the identity, growth and proliferation of cells.^167^

Recently reported lines of evidence indicate that the IDRs of signaling factors in the Wnt/β-catenin pathway are components of condensates that form at SEs in a Wnt-inducible manner.^181^ Moreover, as the pivotal component of the Wnt pathway, β-catenin contributes to the hyperactivation of Wnt signaling via abnormal phase separation induced by IDR-IDR interactions at SEs;^182,183^ this hyperactivation of Wnt signaling is one of the early events in the carcinogenesis of diverse cancers. For instance, almost all colorectal cancers (CRCs) exhibit a hyperactivated Wnt pathway,^184,185^ and the suppression of Wnt signaling can significantly restrict CRC initiation and extend survival.^186^ However, in the absence of Wnt ligands, the multiprotein destruction complex suppresses Wnt signaling and requires the activation of downregulated pathways.^187^ The destruction complex comprises the tumor suppressor APC, the scaffold AXIN, and two kinases—GSK3 and CK1. Recent studies have demonstrated that AXIN and APC undergo LLPS, which is critical for their function in inhibiting the Wnt/β-catenin pathway.^187,188^ Hyperactivation of the Wnt pathway caused by APC mutations is prevalent in a large portion of human CRCs, and these mutants play a pivotal role in the initiation of tumorigenesis by influencing cell differentiation and facilitating rapid proliferation.^189,190^ The above-described examples strongly demonstrate that the regulation of Wnt signaling pathway hyperactivation can be performed in two directions: inhibiting the phase separation induced by Wnt signaling or promoting the LLPS of the destruction complex.

The TF MYC, which has potent cell growth- and proliferation-promoting metabolic activities, is unleashed by genetic and epigenetic dysregulation in cancer.^191^ Mutations or translocations of overactivated enhancers adjacent to the MYC gene are correlated with the pathogenesis of malignancy.^192^ In numerous cancer cells, excessively large SEs have been observed in the gene desert near the MYC gene but are rarely found in their healthy counterparts.^167^ For instance, in multiple myeloma (MM), malignant cells often contain a translocation in which the 3′ IgH SE is inserted near the MYC locus, which drives upregulation of MYC expression^167,193^ (Fig. 3c). In another example, the SE-enriched transcriptional coactivators BRD4 and MED1 are phase-separated into condensates at SEs, which leads to an effectively compartmentalized and assembled transcriptional apparatus.^134^ Indeed, some evidence suggests that BRD4 might mediate transcriptional addiction to the MYC oncogene.^132^ The intervention of MM tumor cell proliferation with the BET-bromodomain inhibitor JQ1 causes a specific reduction in BRD4 at SEs, which results in disruption of MYC transcription elongation.^193,194^ Moreover, BRD4 knockdown can induce cell apoptosis and inhibit the growth of MYCN (a member of the MYC family)-amplified neuroblastomas.^195^ Zhang et al. showed that the focal amplification of SEs in the 3’ direction relative to MYC in lung adenocarcinoma and endometrial cancer are physically connected to the MYC promoter and are correlated with MYC gene overexpression.^178^ SEs not only activate the transcription of protein-coding genes but also regulate the transcription and maturation of noncoding genes, such as miRNAs^196^ and lncRNAs.^197^ Studies have also observed reduced activity of SEs in certain tumor cells. That is, SEs activated in the process of cell carcinogenesis are often related to cancer-promoting miRNAs, whereas inactivated SEs mainly regulate the production of cancer-suppressing miRNAs.^196^

In summary, we realize that SEs can be novel biomarkers useful for the discovery of cancer-specific pathology, and these findings contribute to a deeper understanding of cancer biology, diagnosis, and therapy.^167^

### The deregulated LLPS of tumor suppressors contributes to tumorigenesis

One of the hallmark capabilities of cancer is the evasion of tumor suppressors to limit cell growth and proliferation.^3^ Tumorigenesis is actively inhibited by a spectrum of tumor suppressors, including p53, TGF-β, RB1 and PTEN, which regulate tumor immunology and immune integrity.^198^

#### p53

Among these suppressors, p53 appears to be extremely crucial because it responds to multiple stress signals by coordinating distinct cellular activities, such as permanent and impermanent cell cycle arrest, apoptosis and cell senescence, which are all correlated with tumor suppression.^199–203^ p53 is a cellular stress sensor that suppresses tumorigenesis via transcriptional activation,^204^ and 53BP1 helps to stabilize and enhance p53 gene expression.^199^

However, p53 is mutated in more than 50% of all human cancers^205^ and is functionally inactivated by mutational, viral, or cellular patterns in most types of cancer.^206^ A large proportion of mutant p53 species are highly overexpressed in cancer cells, and some p53 mutations exert negative dominance effects through coaggregation, hetero-oligomerization, and prion-like aggregation with mutant or normal p53 protein. Tumor-associated stress is identified as a strong inducer of p53 aggregation in cell lines.^207–212^ Patients harboring these mutants may have poor clinical outcomes,^212^ which makes these proteins promising therapeutic targets. Increasing studies have attempted to develop methods to inhibit the activity of mutant p53s or to re-establish some wild-type functions that are very promising for cancer therapy.^213^

#### SPOP

The tumor suppressor gene SPOP has gained much attention recently because of its mutation in various cancers.^214^ SPOP forms phase-separated, membraneless clusters in nuclear speckles, and these droplets play central roles in suppressing the tumorigenesis of multiple human malignancies, such as gastric, liver, and prostate cancers.^46,128^ The SPOP droplets function as CRL3 substrate adapters that attract oncogenic substrates for ubiquitination and subsequent proteasomal degradation by a ligase.^128^ Both SPOP self-association and SPOP interactions with substrates can enhance LLPS.^215–217^ When the SPOP substrate protein DAXX is coexpressed with SPOP, another type of liquid-like droplet—SPOP/DAXX bodies—can be formed via LLPS, leading to the ubiquitination of DAXX and thus reducing the DAXX level.^218^ DAXX maintains the survival of cancer cells by downregulating the transcription of various tumor suppressors, including the TF p53^97^ and SMAD4.^93^ DAXX degradation by SPOP effectively induces cancer cell apoptosis and degrades potential therapeutic targets.^219–221^ Moreover, mutations of SPOP are frequently observed in solid tumors, such as breast,^222^ endometrial,^223^ gastric^224^ and prostate cancers,^224,225^ and are associated with early events in tumorigenesis.^218,226^ The consequences of oncogenic mutations in SPOP activities include DAXX recruitment and LLPS disruption, which largely prevents the formation of SPOP/DAXX bodies and thus results in the accumulation of a large number of DAXX proteins.

#### TGF-β

Another tumor suppressor protein, TGF-β, induces growth arrest of cancer cells and repression of the c-MYC proto-oncogene at the early phase.^227^ However, in later stages of malignancy, TGF-β initiates cell invasion and modulates the microenvironment to benefit cancer cell growth.^228^ The interaction of the TGF-β/SMAD and Wnt pathways plays a crucial role in cellular biology, and the opposite function of TGF-β and Wnt is significant for the joint regulation of bone genesis and resorption.^229^ During bone metastasis, the TGF-β pathway maintains disseminated tumor cell dormancy in bone or osteolytic outgrowth.^229,230^ Recently, Esposito et al. demonstrated that TGF-β induces phase separation of the bone metastasis-promoting protein DACT1, which represses the Wnt signaling pathway by sequestering the Wnt pathway activator casein kinase 2. Eliminating the IDRs in the DACT1 protein effectively abolishes its capability to generate liquid-like condensates and the effect of suppression on Wnt signaling.^229^ These findings elucidate the mechanism of cancer bone metastasis and encourage future research toward the inhibition of DACT1 condensate formation.

### Aberrant LLPS interferes with antitumor signaling pathways

As mentioned before, appropriately regulated LLPS can function as a significant hub to promote signal outputs to modulate cellular activities. However, aberrant LLPS is triggered via oncogenic mutations and consequently disturbs the signaling pathways.

Merlin (NF2/schwannomin), another tumor suppressor protein, integrates and regulates intracellular signaling pathways (including the Hippo signaling pathway) and the extracellular matrix and promotes innate immunity against cancer.^25,231^ However, genetic inactivation and mutations of NF2 can be found in a wide range of malignancies, including CRC, schwannomas, type 2 neurofibromatosis, and skin tumors.^232^ Recently, Meng et al. demonstrated that a mutant FERM domain of NF2 potently suppresses the cGAS-STING signaling pathway by forming phase-separated condensates with IRF3 and impedes the antitumor immunity initiated by STING (Fig. 3d).^25^ Clinically, NF2-IRF3 condensates can be observed in surgically resected samples from bilateral vestibular schwannomas.^233^ Therefore, at least in NF2-related malignancies, we can attempt to inhibit the formation of intracellular membraneless structures composed of NF2 to restore the antitumor immunity of the Hippo pathway and cGAS-STING pathway.

In addition, we used to believe that LLPS can only occur in proteins and nucleic acids, but intriguingly, recent studies have shown that other molecules can also generate biomolecular condensates through LLPS and exhibit their own cancer-causing effects. Liu et al. found that the accumulation of glycogen is frequently detected in tumor cells to support increased glucose consumption for tumor growth. The accumulated glycogen can undergo LLPS, leading to the formation of the Laforin-Mst1/2 complex in liver tumors, as shown in Fig. 3f. The Mst1/2 (two Hpo homologs) kinases are significant components of the Hippo pathway in regulating immune systems and cell proliferation via a tumor suppression mechanism.^234^ However, the membraneless Laforin-Mst1/2 complex robustly sequesters the Hippo kinases Mst1/2 and abolishes their repression of oncogenic YAP signaling. Thus, the elimination of glycogen storage can potentially abrogate liver growth and cancer initiation.^235^

Furthermore, a nonreceptor protein tyrosine phosphatase encoded by PTPN11, SHP2, plays an essential role in MAPK signal transduction and organism development.^236^ Disease-associated mutant SHP2 can undergo LLPS and recruit condensates, leading to RAS-MAPK signaling hyperactivation and dysregulation, which is crucial in tumorigenesis events.^237,238^ Researchers have hypothesized that the robust hyperactivation of mutant SHP2 compared with wild-type SHP2 is due to conformational transition. SHP2 allosteric inhibitors effectively inhibit the phase separation of SHP2 mutants and abrogate the capabilities gained by mutated SHP2 droplets, which strongly supports this opinion.^237^

### LLPS of RNA and RBPs in tumorigenesis

In RNA-containing condensates, RBPs often recognize their RNA targets in a specific manner,^239^ and LLPS of RNA with RBPs is facilitated by the distinct properties of the RNA,^240^ such as its identity, length, structure, modifications and expression level. Paraspeckle, a cancer-related biomolecular condensate discovered within the past 20 years,^241^ is a nuclear body constructed via a specific lncRNA—NEAT1_2 RNA—that docks at transcription starting sites of active genes.^72^ A capture hybridization analysis of RNA target experiments demonstrated that NEAT1 RNA is crosslinked to the transcription start sites of active genes in a breast cancer cell line, but the evidence is insufficient to prove that LLPS drives NEAT_2 RNA activity because the probes do not target NEAT1_2 specifically.^242^ In addition, the NEAT1 gene, including the region that encodes NEAT1_2, has been found to be mutated in biopsied samples collected from liver cancer patients,^243^ and hypoxia can trigger the upregulated transcription of NEAT1_2 via hypoxia-inducible factors, which results in a critical upsurge of paraspeckles in breast cancer cell lines.^244^ However, under certain conditions, paraspeckles appear to play tumor-suppressive roles.^245^

Moreover, a study conducted in 2021 showed that the phosphatidic acid-binding lncRNA small nucleolar RNA host gene 9 (SNHG9) facilitates LLPS of LATS1—one of the key kinases of the Hippo pathway—to promote oncogenic YAP signaling.^246^ Clinically, SNHG9 expression was positively correlated with YAP and Ki-67 expression and breast cancer progression.^246^ The role played by RNA in tumorigenesis has been extensively explored by researchers, and we believe that LLPS is an important complement in the causal relationship between multiple RNAs and tumorigenesis.

### Epigenetic dysregulation mediated by LLPS in cancer

Many studies in the field of cancer have concentrated on determinants of genetic alterations that inhibit or facilitate the acquisition of cancer phenotypes. However, a growing body of evidence suggests that destroying certain epigenetic processes can also exert a major impact on cancer development.^247,248^ Epigenetic modifications refer to changes through which cells exhibit a distinct profile of gene expression in identical DNA sequences without irreversibly changing the genetic information.^249^ Among epigenetic modifications involved in regulatory functions, histone modifications, DNA or RNA methylation, chromosome condensation,^116,250^ and lncRNA molecule expression^251^ have been reported to play significant roles in the genesis, progression and metastasis of cancers.^252^

Recent evidence also suggests that EBNA2 undergoes LLPS that reorganizes the host chromatin topology. The N-terminus of EBNA2 mediates the reorganization of chromatin topology via LLPS to induce accessible chromatin domains (ACDs). The CTD of EBNA2 is significant in the epigenetic regulation of host gene expression by recruiting histone acetylase p300 to ACDs, which then mediates the acetylation of certain regions in histone H3K27.^253,254^ Moreover, a study performed in 2019 revealed that histone modifications are essential for maintaining cancer stem cells in human breast cancer. In this study, researchers formed a stem-like breast cancer cell model and observed that the histone deacetylase HDAC7 binds near the transcriptional start site and to SEs of various oncogenes (Fig. 3a) and results in the activation of SE-associated oncogenes.^255^

The most common internal modification of mRNAs, m^6^A, is significantly correlated with gene expression in various cancers.^256^ Recent studies have shown that mRNA modification by multiple m^6^A modifications can function as a multivalent scaffold, promote distinct binding with cytosolic YTHDF proteins and facilitate the LLPS process, which results in the formation of various RNP granules, including P bodies and SGs.^68,257^ P bodies are responsible for RNA decay and storage, and different components of P bodies play different roles in tumorigenesis.^258^ Emerging evidence also demonstrates that abnormalities in the expression and/or activity of SG components contribute to drug resistance and the tumorigenesis of diverse cancers, including CRC,^259^ pancreatic cancer,^260^ and leukemia.^261^ Furthermore, YTHDC1 in the nucleus can undergo LLPS by binding with m^6^A-mRNA (Fig. 3b), and the number of resulting nuclear condensates (nYACs) is greatly increased in acute myeloid leukemia cells.^262^ nYACs protect leukemia-promoting mRNAs from degradation^263^ and maintain cells in an undifferentiated state, which is significant for cell survival and leukemia maintenance.^262^

Therefore, deregulated epigenetic changes may result in tumor cell adaptation and resistance to anticancer therapy, which remain profound challenges to therapeutic intervention,^264^ and targeting the mechanisms of LLPS is a new direction that may lead to elegant strategies for overcoming the complicated situations in cancer therapy.

### LLPS as a strategy for the alternative lengthening of telomeres

**Fig. 6:**
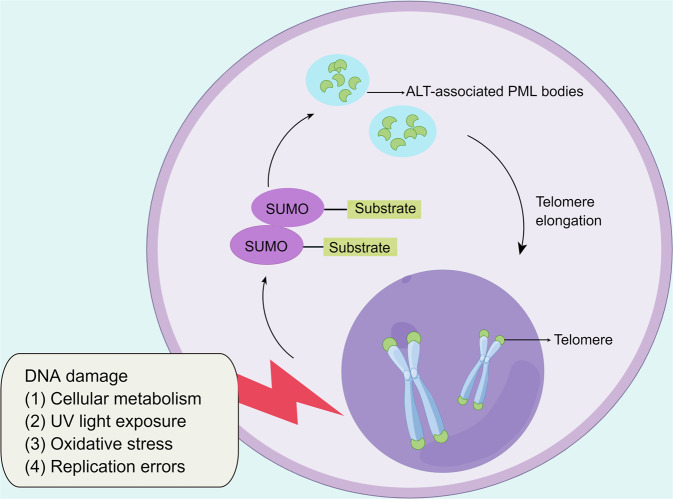
LLPS is a strategy for the alternative lengthening of telomeres. (By Figdraw.). In ALT-related cancer, excessive telomere clustering in PML bodies (ALT-associated PML bodies) can be triggered via poly (SUMO)-poly SUMO interaction motif-mediated LLPS

Telomeres are nucleoprotein structures formed by a repetitive nucleotide sequence (TTAGGG)n that constitutes a “cap structure” at the end of a chromosome, which results in the preservation of genome stability.^265–267^ Telomeres are parts of constitutive heterochromatic regions and are enriched with di- and trimethylated heterochromatin histones,^268^ namely, H3K9me3 and H4K20me3, and characterized by HP1 binding.^269,270^ During eukaryotic cell replication, telomeres continuously shorten, which ultimately leads to cell senescence and apoptosis. In tumorigenesis, tumor cells almost universally acquire telomere DNA maintenance mechanisms (TMMs) that prevent activation of the DDR, which results in counteracting telomere shortening. Two types of TMMs have been recently well characterized: telomerase and alternative lengthening of telomeres (ALTs). In fact, most human tumors exhibit telomerase-based TMM. However, after the development of drugs targeting telomerase, some cancer cells can still escape death, highlighting the less frequently engaged ALT pathway.^267,271,272^ The hallmark of an ALT-related cancer is excessive telomere clustering in PML bodies, which are known as ALT-associated PML bodies (APBs) and are observed as large bright telomere foci.^273^ In response to DNA damage, liquid-like APB formation can be triggered via poly (SUMO)-poly SUMO interaction motif-mediated LLPS (Fig. 6).^274^ The mechanism of telomere lengthening has been shown to be correlated with mitotic DNA synthesis in APB-like foci,^275,276^ and a large quantity of aggregated telomeres at these foci can facilitate mitotic DNA synthesis-mediated ALT.^277^ Some evidence supports the notion that the telomere length is positively associated with the risk of malignancy, such as melanoma, B-cell lymphoma and chronic lymphocytic leukemia.^278–280^

## Prospects of applying LLPS in cancer treatment

Because LLPS can affect tumorigenesis through different pathways, practical strategies for treating these cancer-associated proteins and their upstream/downstream signaling remain to be developed. For the formation mechanisms of biomolecular condensates, we can sustain normal LLPS by controlling the concentration of related proteins/nucleic acids, specifically disrupting the phase separation process, partitioning of cancer drugs in biomolecular condensates, and modifying LLPS by interfering with PTMs, among other strategies.

### Normalizing the protein concentration

Membrane-less condensates assembled from phase-separated proteins are collections of molecules at high concentrations in specific locations, which means that changes in the concentration of relevant molecules can have an impact on the formation and size of LLPS-formed droplets and thus control their function.^134,281^ Therefore, normalizing the protein contents by inducing up- or downregulation of their expression might be a pivotal and valid method for regulating phase separation.

**Fig. 7:**
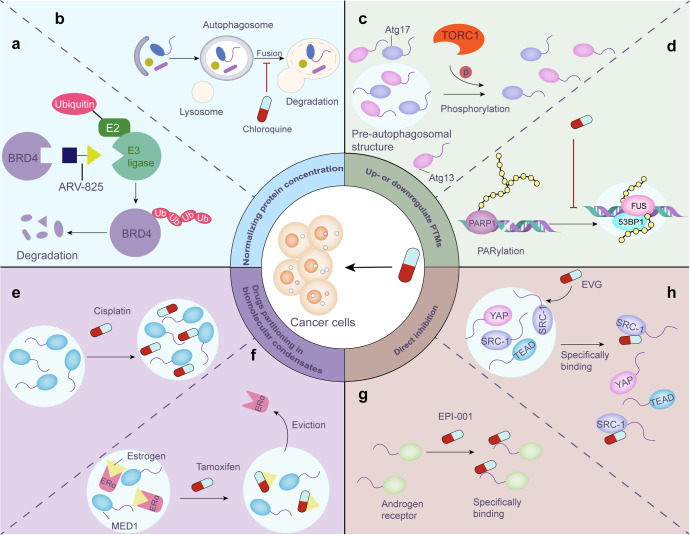
Future prospects of cancer treatment via regulating LLPS. **a** ARV-825, a novel PROTAC, efficiently degrades BRD4 protein in BL cell lines by linking BRD4 to E3 ubiquitin ligase. **b** Chloroquine primarily blocks autophagy by impairing autophagosome-lysosomal fusion, thereby upregulate target protein level. **c** Phosphorylation mediated by activated TORC1 significantly inhibits LLPS and impairs autophagosome prestructure (PAS) formation. **d** Inhibition of PARylation prevents the formation of DNA damage repair foci. **e** Cisplatin preferentially concentrates in biomolecule condensates, which can help improve drug efficacy. **f** Tamoxifen efficiently expels ERα from MED1 condensates via specifically partitioning into these condensates. **g** The small-molecule compound EPI-001 selectively binds to the IDR of the androgen receptor, thereby slowing the progression of castration-resistant prostate cancer. **h** EVG can directly target the SRC-1/YAP/TEAD droplets to restrict cancer cell growth in a YAP-dependent manner

To downregulate the expression of target proteins, an emerging technology referred to as proteolytic targeting chimera (PROTAC) technology can link target proteins to E3 ligases and thus induce their precise degradation in the cell.^282^ PROTACs exploit the cell’s own protein destruction mechanism to clear specific target proteins from cells. Lu et al. designed a heterobifunctional PROTAC, ARV-825, which can lead to efficient and prolonged degradation of BRD4 in BL cell lines by recruiting BRD4 to the E3 ubiquitin ligase cereblon (Fig. 7a)^283^ and thus downregulating the expression of MYC.^284^ In addition, autophagy‐targeting chimera technology is an advanced approach that selectively eliminates target proteins via an autophagy‐dependent pathway.^282,285^

In contrast, to upregulate certain protein contents, preventing their degradation may be a viable option, and in this context, proteasome inhibitors and lysosomal inhibitors have been tested in many trials.^286,287^ For example, chloroquine, an FDA-approved drug that blocks autophagy primarily by impairing autophagosome-lysosomal fusion, has been shown in clinical trials to enhance the potential of combination anticancer therapies by sensitizing tumor cells (Fig. 7b).^287^

However, considering the normal physiological function of the target proteins in different signaling pathways, the specific up- or downregulation of the expression of distinct proteins might be harmful to normal biological activities. Therefore, we need to be careful about the possible side effects when regulating protein expression levels. Detailed clinical trials should be conducted to anticipate side effects and prevent them as much as possible before applying these treatments.

### Induction/inhibition of posttranslational modifications to influence LLPS

PTMs are vital modulators of condensation and affect the properties of membraneless compartments; for example, phosphorylation and methylation of the C-terminal LCD in the fragile X protein, which causes mental retardation, induces contradictory influences on in vitro translation regulation: phosphorylation promotes phase separation and leads to translation inhibition,^288^ and methylation decreases the propensity for LLPS.^289^ Under high nutrient availability, LLPS mediated by the interaction between the autophagy‐related proteins Atg13 and Atg17 is inhibited by phosphorylation via activated TORC1 and then impairs pre‐autophagosomal structure formation (Fig. 7c).^290^ PARylation is another reversible PTM process that is mediated by the catalysis of PAR polymerase (PARP).^291^ The inhibition of PARP1 prevents DNA damage repair foci formation and jeopardizes the process of DDR (Fig. 7d).^130^

### Drug concentrations in biomolecular condensates

Interestingly, researchers have found that some antineoplastic drugs can be selectively concentrated in specific condensates via physicochemical interactions, which may exert curative effects or induce drug resistance in cancer.^292,293^ For example, cisplatin is a widely used antineoplastic agent that partitions selectively with a partition coefficient of 600 in transcriptional condensates where SE DNA can be platinated (Fig. 7e).^294^ This finding reveals the potential of increasing drug target engagement by partitioning the therapeutic components into condensates.

Tamoxifen is another antineoplastic drug that is important for the treatment of estrogen receptor (ER)-positive breast cancer. ERα selectively concentrates into MED1 condensates in an estrogen-dependent manner and is evicted from the condensate by tamoxifen, which also preferentially partitions into transcriptional condensates and competes for binding to estrogen (Fig. 7f). The overexpression of MED1 results in the volume expansion of transcriptional condensates, which results in dilution of the concentrations of tamoxifen in the condensates and counteraction of the efficacy on ERα eviction from the condensate.^294^

Both of these examples provide strong evidence indicating that specific compartmentalization and the concentration of small-molecule cancer therapeutics in condensates can impact drug pharmacodynamics and interventions on phase‐separated complexes and may therefore be efficient for targeting undruggable molecules.

### Drugs directly interacting with biomolecular condensates

Similar to previous expectations, some evidence suggests that certain drugs interact directly with condensates; that is, the application of certain drugs can specifically prevent the formation of condensates that contribute to disease pathology.^294^ Notably, IDRs were preliminarily thought to be undruggable due to their conformational heterogeneity and dynamicity.^295^ However, various recent studies have challenged the identification of different strategies to bind IDRs. For instance, EPI‐001 is a small‐molecule compound that attenuates the progression of castration-resistant prostate cancer by selectively binding to IDRs of the androgen receptor (Fig. 7g).^296^ Moreover, researchers have also analogously demonstrated that the anticancer adjuvant melatonin is capable of inhibiting the intrinsically disordered N-terminal region of prion-mediated phase separation in cancer, which results in the amelioration of multidrug resistance.^297^

Elvitegravir (EVG) was originally developed to treat HIV infection and can potently suppress cancer metastasis by directly targeting the m^6^A methyltransferase METTL3.^298^ EVG can effectively target the SRC-1/YAP/TEAD droplets to restrict cancer cell growth in a YAP-dependent manner by specifically disrupting LLPS of SRC-1 (Fig. 7h).^141^ Recent research has also shown that allosteric regulators may influence LLPS because allosteric sites in intrinsically disordered proteins can be managed to enhance signaling interactions between various mechanistic components.^299^

In addition, interfering with RNA to influence LLPS can be a potential regulatory method. For instance, the RNA helicase DDX3X plays a role in mediating the maturation and disassembly of SGs, and Dhh1 accelerates the aggregation of PBs by linking to Pat1.^300,301^ Furthermore, the molecules that bind to proteins in LLPS, including molecular chaperones and ligands, are important regulatory components.^183,302^

## Concluding remarks and future perspectives

Recent studies have revealed the significance of coordinated actions of biomolecular condensates in orchestrating diverse cellular processes. The driving forces of LLPS are the multivalent interactions between macromolecules accomplished via multiple modular domains, IDRs, and nucleic acid chains. Cutting-edge research on phase separation has successfully reconstituted biomolecular condensates in vitro that simulate the biological features and functions of biomolecular liquid-like droplets in vivo.^37^ Although the IDRs lack a stable 3-dimensional structure, which makes the drug discovery process more challenging, a variety of websites can be used to predict the IDRs in diverse proteins and provide increasingly comprehensive messages of intrinsically disordered proteins.^303–305^ Small-molecule inhibitors directly targeting IDRs have been discovered recently.^306^ For instance, EPI-002 is the first drug tested in a clinical trial that directly binds to the IDR of androgen and shows signs of efficacy in castration-resistant prostate cancer patients.^307^ The analog of EPI-002, EPI-7386, exhibits improved pharmacokinetics and metabolic stability but has never been tested clinically.^307^

When normal LLPS is disrupted by genetic or epigenetic mutations, aberrant biomolecular condensates may be involved in tumorigenesis because of their role in dysregulated chromosome organization, signal transduction, and transcriptional dysregulation and consequently facilitate the development of cancer. Ming et al. demonstrated that cancer cells might be more sensitive and more addictive to LLPS, which suggests the therapeutic potential of LLPS in cancer.^308^ The application of 1,6-hexanediol to pancreatic cancer cells can significantly abrogate the LLPS process and thereby downregulate the expression of the MYC oncogene.^308^

Because LLPS is involved in many cancer mechanisms, various studies have regulated the phase separation process by determining the concentration of related proteins/nucleic acids, directly targeting components that undergo phase separation, or modifying LLPS by interfering with PTMs. In addition, the specific partitioning of antineoplastic drugs in subcellular condensates is also important for drug efficacy because the concentrations of active ingredients can be extremely high in these condensates. According to this characteristic action, we can detect the distribution of anticancer drugs in cells or by linking anticancer drugs to molecules that can specifically aggregate in liquid droplets such that they can directly act on the carcinogenic targets in subcellular condensates.

However, despite the promise of a therapeutic target through LLPS interference, it must be remembered that IDRs are widely distributed in the human body. More than 30% of the regions in the proteome are disordered,^309^ which suggests that we still face enormous challenges in determining the specificity of drug action. We encourage the development of more cell models and animal models to explore the development of anticancer drugs that modulate LLPS, and we hope that more clinical evidence will confirm our conjecture. We believe that joint explorations conducted by cancer researchers, cell biologists, and biophysicists can uncover the mystery of cancer phase separation and can identify more tumor treatment strategies related to phase separation.
